# Blockchain Technologies: Probability of Double-Spend Attack on a Proof-of-Stake Consensus

**DOI:** 10.3390/s21196408

**Published:** 2021-09-25

**Authors:** Mikolaj Karpinski, Lyudmila Kovalchuk, Roman Kochan, Roman Oliynykov, Mariia Rodinko, Lukasz Wieclaw

**Affiliations:** 1 Department of Computer Science and Automatics, University of Bielsko-Biala, 43-309 Bielsko-Biala, Poland; rkochan@ath.bielsko.pl (R.K.); lwieclaw@ath.bielsko.pl (L.W.); 2 IOHK, Singapore 049908, Singapore; lyudmila.kovalchuk@iohk.io (L.K.); roman.oliynykov@iohk.io (R.O.); mariia.rodinko@iohk.io (M.R.); 3 Institute of Physics and Technology, National Technical University of Ukraine “Igor Sikorsky Kyiv Polytechnic Institute”, 03056 Kyiv, Ukraine; 4 Department of Computer Science, V.N. Karazin Kharkiv National University, 61022 Kharkiv, Ukraine

**Keywords:** blockchain, consensus protocol, proof-of-stake, fork, double spend attack

## Abstract

Two double-spend attack strategies on a proof-of-stake consensus are considered. For each strategy, the probability of its success is obtained, which depends on the network parameters and the number of confirmation blocks. These results can be used to define how many confirmation blocks a vendor should wait after a correspondent transaction before sending goods or services.

## 1. Introduction

Since Bitcoin launched in 2009, blockchain systems and distributed ledger technologies have become popular, received widespread adoption, and attracted significant research effort [1].

They provide a great use case and have big advantages in environments that require no trust. That includes various financial applications (DeFi), cryptocurrencies, different types of distributed registries, etc. The decentralized blockchain-based systems provide the common view on the history of transaction ledger, censorship resistance, and no single point of failure.

Although, comparing to the centralized approach, a decentralized environment has much longer latencies on transaction confirmations. As there is no central server(s), the majority of network nodes in a trustless environment should receive a new transaction, validate it, and share that with other nodes, working in conditions with delayed message delivery over the network.

Moreover, some part of network participants may be well coordinated by an adversary who attacks the system. At the same time, honest nodes have no ability to discern the malicious behavior until an attack is finished (with any result).

Within such conditions, a distributed system user must decide whether she accepts the transaction (and provides corresponding services or goods for the accepted value) or she should wait for higher confirmation assurance (or just reject the transaction).

For many practical applications, such as on-line exchanges or retail, it is critical to minimize the confirmation time latency.

So, on given input parameters (such as the adversarial ratio among all nodes reaching consensus), it is important to define concrete criterion regarding when a transaction may be secure and accepted with low risk and when it should be removed from the final history of the blockchain.

Thus, the special case of persistence, as one of two major ledger properties [2], needs to be analyzed: the resistance to the double-spend attack. The essence of this attack does not depend on the type of consensus protocol. Technically, it happens as follows. An adversary carries out some transaction in the block with number *i*, transferring coins to a supplier of goods for some purchase. The supplier receives those coins and accordingly supplies the goods to the buyer. The adversary also starts mining (forging) a different block with the same height *i*—that is, a block following the block with number (*i*–1), but one that either does not contain this transaction—or he transfers coins to another one of his own accounts. To guarantee acceptance of this alternative chain by honest participants, he tries to “hook” as many blocks as possible to the alternative block at height *i*. If he succeeds in making the alternative chain longer, then exactly this chain, according to the consensus protocol, will be the one that is considered correct. Obviously, the larger the share kept by the adversary (it is not essential whether is it computing power in the case of PoW or a share of a stake in the case of PoS), the higher chance his attack has of being successful. In particular, if the share of the adversary is not less than 1/2, then the probability of the attack success is equal to 1.

A double-spend attack may seem to be very similar to a selfish mining attack, but they have two main differences. The first difference consists in their purposes: the purpose of a double-spend attack is to use the same coin for two (or more) different payments, creating conflicting transactions; the purpose of a selfish mining attack is to generate essentially more blocks than is expected for a given adversary’s ratio and, therefore, to get profit that is not proportional to his share. The second difference yields from the first one: in the double-spend attack, an adversary should keep his alternative branch in secret until the necessary number of confirmation blocks are created; therefore, he builds this chain only by himself, while in selfish mining, honest miners see the alternative chain and occasionally may maintain its creation.

In this work, we investigate only the security of the general model of PoS protocol against a double-spend attack under only one assumption, which is quite standard: the probability that the next block is generated by some stakeholder is proportional to its stake. Our innovative contribution is that we for the first time obtain formulas for exact values of probability of a double-spend attack, unlike lots of previous papers, which give only asymptotic estimations of such probabilities or their upper bounds, which for some sets of parameters are trivial (i.e., take values that are ≥1). Informally speaking, such papers state: “if you are waiting for an infinitely long time, the probability that an adversary may invert your transaction is infinitely small”. However, nobody wants to wait an infinitely long time; the vendor wants to know exactly how many confirmation blocks he should wait to be sure (with some predefined probability) that transaction is irreversible. Our results are just for such case—for an arbitrary ratio of adversaries and numbers of confirmation blocks, the vendor can achieve the following:

(1) For a given number of confirmation blocks, calculate the probability that his transaction is irreversible; or
(2) Set some desirable level of probability (say, 0.999) and calculate the minimal number of confirmation blocks he should wait to be sure that his transaction is irreversible.

A more detailed comparative analysis between our results and previous works will be given in the next section.

We also give a lot of examples of numerical results, which were obtained according our formulas, and corresponding graphs. They also confirm the correctness and practical benefits of the statements and formulas obtained in this work.

## 2. Related Work

The first mention of a double-spend attack and its detailed description was made by Nakamoto in his historical paper [3].

To ensure protection against this attack in Bitcoin, Nakamoto proposed not to supply the goods as soon as the transaction occurred, but to wait for some time, more precisely for a number of confirmation blocks, and only then, if the transaction has not disappeared from the blockchain, to supply the goods. In this case, the adversary cannot open his alternative chain immediately after the payment, as then the provider will see that the transaction disappears and then appears in the blockchain and thus reject the transaction. For this reason, the adversary first waits until the block with the transaction “grows” by the required number of confirmation blocks. During this waiting period, he can try to seamlessly generate a fork that starts before the block with the transaction; that is, in our notations, he may generate an alternative *i*th block with the blocks to follow, but in no case does he share this alternative chain during the confirmation period, so that the supplier will not suspect anything bad. This is the first stage of the attack. However, when the confirmation blocks are formed and the goods are received, the adversary tries to “catch up” with the existing chain, and this is the second stage of the attack. Suppose that while six confirmation blocks are being generated, the adversary was able to generate four blocks of the alternative chain. Now, he lags behind by at least two blocks. If ever in the future he is able to generate as many blocks as it is needed to “catch up” with the existing chain, which, in turn, will also grow all the time, then the attack will be successful. In particular, if he managed to generate seven or more blocks at the first stage of the attack while he waited for the confirmation blocks, then the attack was already successful: there is nothing to catch up. Having received the goods, he simply presents his own longer chain, in which the money remains with him.

Now, the next question is: how many confirmation blocks should the supplier wait? In other words, for the given network parameters and given (arbitrarily small) ε>0, what number of confirmation blocks after a transaction should he wait for a probability of a successful attack to be smaller than that given ε?

The answer to this question, given in [3] by Nakamoto and in [4] by Saleh, requires correction. The assumptions made in [3] do not quite correspond to the real deployment model. The first assumption is that the time of generation of the block and the time of its appearance in the network coincide, so the block propagation delay is zero. However, from this assumption, it follows that the probability of an “accidental” fork is zero, but reality shows that such forks happen [5]. The second assumption states that the random variable, which is equal to the number of attempts that honest miners do to generate z confirmation blocks, where p is the probability of success, is replaced by its expectation $\frac{z}{p}$. Due to these assumptions, the number of confirmation blocks in [3] is underestimated.

After [3], the probability of a double-spending attack was analyzed in papers [6,7], which also have some lacks, including unproved statements. For the first time, the problem gets a fully correct solution in [8], which is really wonderful from the mathematical point of view. In this paper, the authors prove that the process of generating “honest”/”dishonest” blocks in the network is described by a negative binomial distribution. It was first proved in this paper, using special functions, that the fork probability decreases exponentially with the growth of the number of confirmation blocks.

However, the authors of [8] could not and even did not try to get rid of the same assumption on the instantaneous propagation of the block in the network.

The work [9] generalized the results obtained in [8]. In this work, for the first time (in model with continuous time, without simplified assumptions about discrete timeslots), the author obtained and strictly proved the expression that gives the value of double-spend attack probability in dependence on network parameters, including network synchronization time.

Note that all these works [3,4,6,7,8,9] investigate proof-of-work consensus protocol, but there are still no analogical results for proof-of-stake [10,11,12]. However, consensus protocol proof-of-stake is much more preferable than proof-of-work from a lot of points of view. The main problems that occur on block generation with PoW consensus are the following:

− Miners must be on-line and continuously solve PoW puzzles;
− Huge energy consumption to generate a block with an acceptable security level;
− Occasional forks where parts of work made by honest miners are lost.

To solve these problems, as well as several other ones, a proof-of-stake-based approach was proposed. The first provable secure PoS was presented in [13] as well as its next generations [14,15,16,17].

The main idea of PoS consensus is randomized slot leader selection; i.e., a participant who forges the next block is randomly selected in a non-biased way to issue the block within a given period of time. The probability to become a slot leader is proportional to the stake owned by the participant. A detailed description of PoS approaches is given in [13,14,15,16,17,18,19], as well as definitions, a model with strict formalizations, and security proofs.

For the first time, the rationale for the robustness of PoS protocol was given in [13] under the standard assumption that slot leaders are chosen among stakeholders with probabilities that are proportional to their stakes. To assure such an assumption, an Ouroboros protocol was proposed, which was modified and improved in the next papers [13,14,15,16]:

- Ouroboros (Classic) [13]—the first provable secure PoS consensus protocol;
- Ouroboros Praos [14]—security against fully-adaptive corruption in the semi-synchronous model;
- Ouroboros Genesis [15]—security with a dynamic participation model;
- Ouroboros Chronos [16]—a provable secure PoS consensus protocol that is independent of global time.

The statements formulated about PoS security in [13] are “general”; they concern such properties of protocol as “liveness” and “persistence”. Informally speaking, the deeper the block, the higher the probability that it is stable. Most of the statements about block stabilization given in [13] are upper estimations of probabilities (which sometimes turn out to be trivial for certain values of parameters) or descriptions of their asymptotic behavior. Such results persuade us that the probability of block stabilization increases fast when the depth of the block increases, but it cannot be used to calculate the minimal number of confirmation blocks after which we are sure that the block is stable.

A lot of papers published every year analyze different additional properties and applications of PoS protocols. Among others, we can note [20], where the authors combine PoS protocol with secure BTC blockchain to obtain a consistent subchain; [21] analyzes the liveness of sidechains, built on PoS, using a special multisignature; [22] discusses PoS with a digital signature scheme that prevents the validators from creating multiple blocks at the same height; [23] considers two cases of smart-contracts of blockchain with PoS; [24] is also devoted to the use of smart-contracts on a private Ethereum blockchain. These works analyze some special aspects of PoS security, but none of them give the answer on such a simple, practical, and specific question: how many confirmation blocks is enough to guaranty block stability with a given probability?

In our paper, we provide analytical estimations of a double-spend attack in the covert adversary model for an arbitrary version of Ouroboros protocol.

Our estimations are strict (not asymptotic), which allows using them to define a necessary number of transaction confirmation blocks that is sufficient to make the transaction irreversible with any given probability (e.g., 1–10^−3^).

It is interesting that the estimations obtained in this work for PoS protocol are very close to the corresponding results for PoW protocols, which were firstly obtained with a full mathematical background in [8]. However, for obtaining such estimations, rather different probabilistic methods were used (e.g., random processes with continuous time for PoW and random sequences for PoS).

Our analytical estimations allowed obtaining concrete values of the confirmation of block numbers depending on system parameters (including adversarial stake participating in consensus) and building dependence diagrams for them.

## 3. Materials and Methods

In this section, we describe two possible strategies for the implementation of a double-spend attack. The first one is more universal; it is suitable for almost any consensus protocol. It was first proposed by Nakamoto in his historical work [3] for PoW consensus protocol. The second one is specific for the PoS (and, may be, for some limited class of other) protocol. For both strategies, we give exact analytical expressions for the probability of success of the attack, which depends only on an adversary’s ratio and the number of confirmation blocks that the vendor should wait before sending goods. We stress that these expressions are non-asymptotical, so they may be also used for such purpose as to find the minimal number of confirmation blocks, which guarantees that the attack probability is less than some predefined small value, such as $\varepsilon =10^{-3}$ or less.

Note that the security of some object, model, system, or process against any attack is defined by two main parameters: the computational efforts (or/and, maybe, the volume of memory) needed for the successful implementation of this attack and probability of its success. If the adversary needs unreachable computational efforts to implement the attack with significant probability during some appropriate period of time, or if the probability of attack is negligible, we say that this object (model, system process, etc.) is secure against this attack.

In the model of a double-spend attack discussed in our work, we make some assumptions in favor of the adversary: we assume that he has unlimited time to implement the attack. Recall that the adversary’s ratio, which is defined by his stake, is minority, i.e., less than 50%.

In this section, we are going to prove the next result: for arbitrary adversary’s ratio of $0\leq q<\frac{1}{2}$ and arbitrarily small ε > 0, there is such z = z(*q*, ε) that after z confirmation blocks the probability of a double-spend attack, which is implemented according to the strategies described below, is less than this given ε. It means that the vendor can reduce the probability of a double-spend attack to an arbitrarily small and negligible value, waiting for some certain number of confirmation blocks. In other words, in such a manner, we can achieve an arbitrarily large level of security of PoS protocol against a double-spend attack, even in the model when the adversary has unlimited time (but a minority of stake).

In what follows, we will use the next designations. Let $B_{0},\;B_{1},\;\ldots \;,\;B_{n}$ be blocks of the blockchain, and let some transaction with a payment for the vendor be included into block $B_{i}$, for some i∈N. Then, the vendor waits until z blocks have been linked after this block to be sufficiently certain the sender cannot prune it.

At the same time, the adversary (sender) wants to prune the block $B_{i}$ with his transaction and take money back.

In this model, the adversary tries to organize a branch point in block $B_{i-1}$, just before the block with the transaction. We will consider two different strategies of the adversary.

### 3.1. Strategy 1: Description of Attack and Estimation of Probability of Its Success

The adversary does not form his blocks in his timeslots in the chain that honest miners build. Instead, he starts to form an alternative branch with a branch point (BP) in $B_{i-1}$, where block $B_{i}$ is pruned: $B_{1},\;\ldots \;,\;B_{i-1},\;B_{i}^{\prime },\;B_{i+1}^{\prime },\;\ldots \;$. After z blocks $B_{i+1},\;B_{i+2},\;\ldots \;,\;B_{i+z}$ are formed, he tries to make his alternative branch longer.

Note that according to this strategy (during the attack, the adversary does not form his blocks in the”honest” branch) all blocks in the chain, starting from $B_{i-1}$, are formed by honest parties.

To succeed, the adversary must build an alternative chain that is longer than the “honest” one. It is possible only in case if, in some timeslot with number s, after the blocks $B_{i+1},\;B_{i+2},\;\ldots \;,\;B_{i+z}$ are formed, the number of “adversary’s” timeslots between the timeslot that corresponds to the block $B_{i-1}$ (let it be the timeslot with the number t) and the timeslot with number s, is not less than the number of “honest” timeslots on the same time interval. In this case, the adversary can form the alternative chain:

$$B_{1},\;\ldots \;,\;B_{i-1},\;B_{i}^{\prime },\;B_{i+1}^{\prime },\;\ldots \;,$$

for some r, where all blocks are formed in his timeslots, and block $B_{r}^{\prime }$ is formed in timeslot number s.

Hence, the necessary and sufficient condition for the successful attack is the existence of such a sequence of timeslots after the timeslot with number t, where the number of “malicious” slots is not less than the number of “honest” slots.

To find the probability for successful attack, we will use the random excursions model (REM) [12].

Firstly, we need to formalize the problem using REM.

We assume that among n parties, t are malicious (t<n2) and n−t are honest. So, the probability that an arbitrary timeslot is honest is p=(n−t)n, and the probability of a malicious timeslot is q=tn.

Let $\xi_{i},i\geq 1$ be a Bernoulli random variable (RV),

(1) $$\xi_{i}=\left\{ \begin{array}{l} -1,\;\;with\;\;probability\;\;q,\\ 1,\;\;with\;\;probability\;\;p. \end{array}\right..$$

Here, a random sequence $\xi_{i},i\geq 1$ describes the distribution of timeslots in the blockchain. If $\xi_{i}=1$, then the slot leader of the *i*th timeslot is honest; if $\xi_{i}=-1$, then he is malicious.

Let us define the following random variables:

(2) $$S_{0}=0,\;S_{n}=\sum_{i=1}^{n}\xi_{i},$$

$$S_{0}^{+}=0,\;S_{n}^{+}=\sum_{i=1}^{n}\left(\xi_{i}\vee 0\right),$$

(3) $$S_{0}^{-}=0,\;S_{n}^{-}=\sum_{i=1}^{n}\left(-\xi_{i}\vee 0\right).$$

The physical senses of these random variables are the following:

- $S_{n}^{+},n=0,1,\ldots$ is equal to the number of timeslots that the honest slot leader has on the interval between the slot with the number 0 and the slot with the number *n*;
- $S_{n}^{-},n=0,1,\ldots$ is the analogical value for the number of the adversary’s slots;
- $S_{n},n=0,1,\ldots$ is equal to $S_{n}^{+}-S_{n}^{-}$; i.e., the difference between “honest” and “malicious” slots.

In this model, we consider a random excursion that starts at the moment t, so there are z+1 “honest” blocks after it, before the adversary tries to build an alternative branch. He can use only timeslots after block $B_{i-1}$, because this block is a branch point (BP).

Fix some k∈N and let us define a new random variable

$$\tau_{k}=\min\left\{l\geq 1:S_{l}^{+}=k\right\}.$$

Here, $\tau_{k}$ is the number of timeslots, such that on the interval $\left[0,\tau_{k}\right]$, there exists exactly *k* slots, which belong to the honest slot leaders.

Now, in this definition, our purpose is to find the probability of the event

$$A\left(k\right)=\left\{\exists m>\tau_{k}:S_{m}^{-}\geq S_{m}^{+}\right\},$$

for k=z+1, where $S_{m}^{-},\;\;S_{m}^{+}$ are defined according to (1)–(3).

The event *A*(*k*) is just the event that after *k* confirmation blocks were built, at some moment, the adversary managed to build the longer chain using his timeslots.

In what follows, we need the result in a random excursion, “gambler ruin problem” [25]. We formulate this result as the next lemma.

**Lemma** **1.** ([25]). *In designations (1)–(3), let us define*

$$S_{n}^{\left(k\right)}=S_{n}+k,\;\;S_{0}^{\left(k\right)}=k.$$

Let $C_{k}$ be the next event: $C_{k}=\left\{\exists l\in N:S_{l}^{\left(k\right)}=0\right\}$, and $q_{k}$ be its probability: $q_{k}=P\left(C_{k}\right)$. Then

$$q_{n}=\left\{ \begin{array}{l} 1,\;\;\;if\;\;q\geq p,\\ {\left(\frac{q}{p}\right)}^{k},\;\;else \end{array}\right..$$

Informally speaking, this lemma states that if at some moment an adversary with a stake ratio *q* is *k* blocks behind, the probability that he will catch up (during unlimited time) is ${\left(\frac{q}{p}\right)}^{k}.$

To prove our main result, we also need some definitions and properties of special functions [6,7]. The matter is that the probability of this attack can be expressed in terms of a well-known special function: a regularized incomplete beta function. Now, we give its definition and main properties that are used in the statements presented below.

**Definition** **1.** *Regularized incomplete beta function is the function.*

(4) $$I_{x}\left(a,b\right)=\sum_{l=a}^{\infty }C_{b+l-1}^{l}x^{l}{\left(1-x\right)}^{b}=\frac{B_{x}\left(a,b\right)}{B\left(a,b\right)},$$

*where* $B_{x}\left(a,b\right)=\int_{0}^{x}t^{a-1}{\left(1-t\right)}^{b-1}dt$ *is an incomplete beta function,*

$B\left(a,b\right)=B_{1}\left(a,b\right)=\int_{0}^{1}t^{a-1}{\left(1-t\right)}^{b-1}dt=\frac{\Gamma \left(a\right)\Gamma \left(b\right)}{\Gamma \left(a,b\right)}$ *is a beta function,*

$\Gamma \left(x\right)=\int_{0}^{\infty }t^{x-1}e^{-t}dt$ *is a gamma function.*

**Lemma** **2.** ([26]). *The regularized incomplete beta function satisfies the symmetry relation:*

$$I_{p}\left(a,b\right)+I_{q}\left(a,b\right)=1\;\mathrm{for}\;0\leq p,q\leq 1,\;\;\;p+q=1.$$

From Lemma 2 and definition of inverse binomial distribution, we get the next corollary.

**Corollary** **1.** *In our designations,*$\sum_{l=0}^{z}C_{z+l}^{l}q^{z+1}p^{l}=\sum_{l=z+1}^{\infty }C_{z+l}^{l}p^{z+1}q^{l}$.

Now, we are ready to formulate the next theorem.

**Theorem** **1.** *In our designations, the next equality for probability of a double-spend attack after z confirmation blocks is true:*

(5) $$P\left(A\left(z+1\right)\right)=2\sum_{l=0}^{z}C_{z+l}^{l}p^{l}q^{z+1},$$

*or using the local Moivre–Laplace theorem, for appropriate* p *,* q *and* z *:*

(6) $$P\left(A\left(z+1\right)\right)=2p\sum_{l=0}^{z}\frac{\varphi \left(\frac{zq-lp}{\sqrt{\left(z+l\right)pq}}\right)}{\sqrt{\left(z+l\right)pq}},$$

*or using a regularized incomplete beta function:*

(7) $$P\left(A\left(z+1\right)\right)=2I_{q}\left(z+1,z+1\right),$$

*and, finally, for sufficiently large* z *:*

(8) $$P\left(A\left(z+1\right)\right)=O\left({\left(4pq\right)}^{z+1}\right).$$

**Proof** **of Theorem 1.** Define the events:

$$H_{l}=\left\{\tau_{z+1}=z+1+l\right\}=\{S_{\tau_{z+1}}^{-}=l\},\;l\in \left\{0,1,\;\;\ldots \right\}.$$

Event $H_{l}$ means that the adversary accumulated exactly l blocks before the time when slot $\tau_{z}$ begins.

Note that $H_{l}$, l∈{0,1,  …}, forms the full group of events.

Then, according to the composite probability formula:

(9) P(A(z+1))=∑l=0∞P(A(z+1)Hl)P(Hl).

The probabilities of $H_{l}$, l∈{0,1,  …}, have a negative binomial distribution:

(10) $$P\left(H_{l}\right)=C_{z+1+l-1}^{l}p^{z+1}q^{l}=C_{z+l}^{l}p^{z+1}q^{l},$$

where

(11) $$\sum_{l=0}^{\infty }C_{z+l}^{l}p^{z+1}q^{l}=1.$$

According to Lemma 1,

(12) P(A(z+1)Hl)={(qp)z+1−l,  when  q<p  and  l<z+1;1,   else  (when  q≥p  or  l≥z+1)..

Then, rewrite (9) using (10)–(12) and obtain:

(13) $$\begin{array}{l} P\left(A\left(z+1\right)\right)=\sum_{l=0}^{z}C_{z+l}^{l}p^{z+1}q^{l}{\left(\frac{q}{p}\right)}^{z+1-l}+\sum_{l=z+1}^{\infty }C_{z+l}^{l}p^{z+1}q^{l}=\\ =\sum_{l=0}^{z}C_{z+l}^{l}p^{z+1}q^{l}+\sum_{l=z+1}^{\infty }C_{z+l}^{l}p^{z+1}q^{l}=1-\sum_{l=z+1}^{\infty }C_{z+l}^{l}p^{z+1}q^{l}+\sum_{l=z+1}^{\infty }C_{z+l}^{l}p^{z+1}q^{l} \end{array}$$

From the definition 1, Formula (4), Lemma 2, Corollary 1, and (13), we obtain:

$$P\left(A\left(z+1\right)\right)=1-I_{p}\left(z+1,z+1\right)+I_{q}\left(z+1,z+1\right)=2I_{q}\left(z+1,z+1\right)=$$

$$=2\sum_{l=0}^{z}C_{z+l}^{l}q^{z+1}p^{l},$$

and Formulas (5) and (7) are proved.

To prove (6), for appropriate z, p, and q (i.e., when zpq>25 or when p≤0.9 and npq>25), we rewrite $C_{z+l}^{l}p^{z+1}q^{l}$ as $p\cdot C_{z+l}^{l}p^{z}q^{l}$ and use the local Moivre-Laplace theorem [25] for (5):

$$C_{z+l}^{l}p^{z}q^{l}=\frac{\varphi \left(\frac{zq-lp}{\sqrt{\left(z+l\right)pq}}\right)}{\sqrt{\left(z+l\right)pq}},$$

where φ(x) is the standard normal distribution density, $\varphi \left(x\right)=\frac{e^{-\frac{x^{2}}{2}}}{\sqrt{2\pi }}$.

To prove (8), note that [26]

$I_{q}\left(z+1,z+1\right)=\frac{1}{2}I_{4q\left(1-q\right)}\left(z+1,\frac{1}{2}\right)=\frac{1}{2}I_{4qp}\left(z+1,\frac{1}{2}\right)$, when $0\leq q\leq \frac{1}{2}$, and for fixed x, b (b>0,  0<x<1) and for a→∞, for each n=0,1,  …, the next equality is true:

$$I_{x}\left(a,b\right)=\Gamma \left(a+b\right)x^{a}{\left(1-x\right)}^{b-1}\left(\sum_{k=0}^{n-1}\frac{1}{\Gamma \left(a+k+1\right)\Gamma \left(b-k\right)}{\left(\frac{x}{1-x}\right)}^{k}+O\left(\frac{1}{\Gamma \left(a+k+1\right)}\right)\right)$$

So, for n=0, we obtain:

$$P\left(A\left(z+1\right)\right)=2I_{q}\left(z+1,z+1\right)=I_{4pq}\left(z+1,\frac{1}{2}\right)=$$

$$=\Gamma \left(z+1.5\right){\left(4pq\right)}^{z+1}{\left(1-4pq\right)}^{-\frac{1}{2}}\times O\left(\frac{1}{\Gamma \left(z+2\right)}\right)=O\left({\left(4pq\right)}^{z+1}\right)$$

The theorem is proved. □

### 3.2. Strategy 2: Description of Attack and Estimation of Probability of Its Success

In this strategy, the adversary forms his blocks in the same way that honest parties do all the time and issues them to both “honest” and “malicious” chains, but his purpose is the same: to choose some appropriate moment for building a longer alternative chain with the branch point in $B_{i-1}$ and with block $B_{i}$ without his transaction.

As in the previous case, in this alternative chain, he may use all his timeslots after the slot in which $B_{i-1}$ was formed. The main difference is that all blocks in this chain are formed in the successive timeslots without missing, i.e., to form z “honest” blocks after $B_{i}$, we need just z timeslots.

To make the alternative chain with a branch point in $B_{i-1}$, the adversary can use all his timeslots after (i−1)th (in which $B_{i-1}$ was formed). Then, on the interval from $B_{i}$ to $B_{i+z}$, he may have from 0 to (z+1) timeslots.

We will use the same REM, defined in (1), (2), with q<p,  q+p=1.

For some k∈N, define the event: $E\left(k\right)=\left\{\exists \;m\geq k:S_{m}^{-}\geq S_{m}^{+}\right\}$.

We are interested in finding the probability P(E(z+1)).

**Theorem** **2.** *In our designations, the probability of a double-spend attack, according to Strategy 2 and after z confirmation blocks, is:*

(14) $$P\left(E\left(z+1\right)\right)={\left(2q\right)}^{z+1}.$$

**Proof of Theorem** **2.** Let us define the events:

$$H_{l}=\left\{S_{z+1}^{-}=l\right\},l=\bar{0,z+1}$$

Event $H_{l}$ means that the adversary accumulated l timeslot between timeslot $t_{1}$, which corresponds to block $B_{i-1}$, and timeslot $t_{2}$, which corresponds to block $B_{i+z}$.

Note that $H_{l},l\in \left\{0,1,\;\ldots \right\}$ forms the full group of events.

Then, according to the composite probability formula:

(15) P(E(z+1))=∑l=0z+1P(E(z+1)Hl)P(Hl).

Probabilities of $H_{l},l\in \left\{0,1,\;\ldots \right\}$ are the probabilities of binomial distribution:

(16) $$P\left(H_{l}\right)=C_{z+1}^{l}q^{l}p^{z+1-l},\;l=\bar{0,z+1}.$$

Probabilities of P(E(z+1)Hl) can be obtained according to Lemma 1:

(17) P(E(z+1)Hl)={(qp)z+1−l,  when  q<p  and  l≤z+1;1  ,   else..

Then, rewrite (15) using (16) and (17) and obtain:

$$P\left(E\left(z+1\right)\right)=\sum_{l=0}^{z+1}C_{z+1}^{j}q^{l}p^{z+1-l}{\left(\frac{q}{p}\right)}^{z+1-l}=\sum_{l=0}^{z+1}C_{z+1}^{l}q^{z+1}\;=q^{z+1}\sum_{l=0}^{z+1}C_{z+1}^{l}=q^{z+1}\cdot 2^{z+1}={\left(2q\right)}^{z+1}.$$

The theorem is proved. □

## 4. Results and Discussion

**Figure 1:**
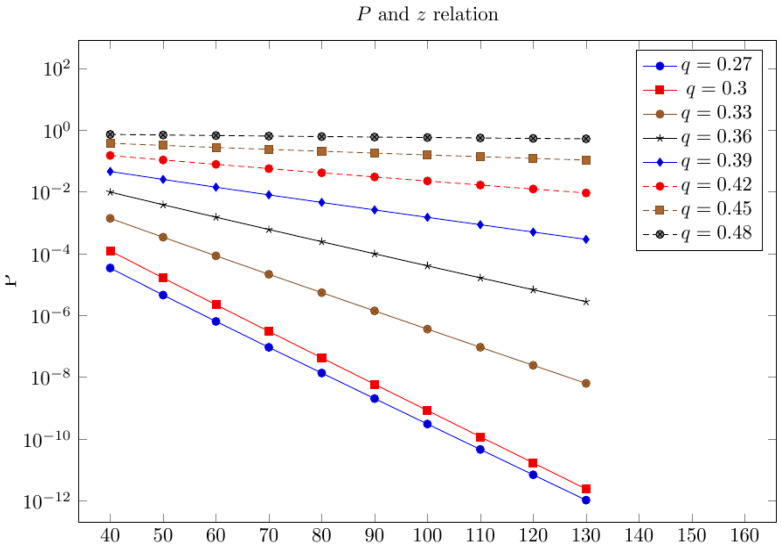
The logarithms of a double-spend attack probability for a different adversary ratio of q.

Figure 1 below gives the dependency of the logarithm value of probability P(A(z+1)) of a double-spend attack under z confirmation blocks, which were obtained in (7) (on the Y-axis), on the value z (on the X-axis), for different adversary ratios. As long as the graphics for the logarithm of probability are straight lines, then the value P(A(z+1)) decreases exponentially with the growth of z. According to the Formula (8), the function decrease rate P(A(z+1)) with the growth of z is the same as for function ${\left(4pq\right)}^{z+1}$.

.

It is interesting that the probability of a double-spend attack in PoS consensus in the synchronous model turns out to be equal to the probability of the same attack in PoW consensus under the assumption of zero synchronization time [8]. However, the methods of obtaining these two results for these two consensuses are essentially different.

Using our results, we can now reasonably recommend to the vendor how many confirmation blocks he should wait after the correspondent transaction before sending goods or services. The only information we need to set is the stake share of the alleged attacker. This information we can get from the different mining pools’ observations and analysis, which shows us that some mining pool(s) behaves suspiciously.

Table 1 below shows some values $P\left(A\left(z+1\right)\right)=2I_{q}\left(z+1,z+1\right)$, according (7), for a different ratio q of the adversary and different numbers of confirmation blocks z.

In Table 2, we show the minimal values of z, for a different ratio q, which provides the condition $P\left(A\left(z\right)\right)<10^{-3}$. It means that if the vendor waits for such a number of confirmation blocks, the probability of a double-spend attack, even during an infinitely large amount of time, is less than $10^{-3}.$

Note that our results once more demonstrate that PoS protocol is more preferable than PoW from the point of view of its security against a double-spend attack. The matter is that usually, the timeslots are large enough (comparing with the synchronization time) so that the slot leader can create a block and share it among other participants during his timeslot. So, we may neglect the network synchronization time and work with a synchronous model, in which the security level against the double-spend attack is defined only by the adversary’s stake ratio. However, in PoW, the synchronization time plays a very important role. As shown in [9,27], the security threshold (the minimal adversary ratio that can implement the attack with probability 1 despite the number of confirmation blocks) decreases when the synchronization time increases, and in the case of a large synchronization time, even an adversary with a minority hashrate can implement the attack with probability 1).

## 5. Conclusions

We researched two types of strategies for a double-spend attack and give strictly proved expressions for success probabilities for both of them. Note that comparing Formulas (7) and (14), we see that Strategy 1 is always more preferable for the adversary than Strategy 2. Indeed, under condition $p>\frac{1}{2}>q$, we get the next inequality:

$$4pq>4\cdot \frac{1}{2}q=2q$$

So, the success probability in Strategy 2 is less than the same probability in Strategy 1 under the same values of q and z.

Using the results obtained, one can define the necessary number of confirmation blocks to make this probability negligible.

There are two interesting questions that we would like to investigate in our next works:

1. To consider an asynchronous model, where the adversary can delay the message delivery for honest slot leaders for some significant period of time, for example, equal to several timeslots, and analyze the probability of a double-spend attack in a such model.
2. To obtain similar results in a case when the adversary has only a limited period of time to implement his attack. Such models occur when, for example, the blockchain has checkpoints, and the adversary can create a fork only in the period before the next checkpoint.

## Figures and Tables

**Table 1:** Double-spend attack probability $P\left(A\left(z\right)\right)=2I_{q}\left(z,z\right)$.

$\frac{q}{z}$	0.10	0.15	0.20	0.25	0.30	0.35	0.40	0.45
5	0.00178184	0.0112573262	0.03916288	0.0978546142	0.19761732	0.343438571	0.53313536	0.7571581092
10	7.85976466 × 10^−6^	0.000288	0.0031582412	0.0178065586	0.0651067138	0.1749472008	0.372184042	0.657928176
15	3.9252264 × 10^−8^	0.00000822	0.000284	0.0035685234	0.023307658	0.095273444	0.2724259	0.58606496
20	2.0678238 × 10^−10^	0.000000248	0.0000268	0.000748	0.008673864	0.053573446	0.20411726	0.52863006
25	1.1224685 × 10^−12^	7.7 × 10^−9^	0.00000258	0.0001606	0.0033027272	0.030712034	0.155151124	0.48059132
30	6.2112992 × 10^−15^	2.44 × 10^−10^	0.000000256	0.000035	0.001276	0.0178373424	0.119104008	0.439334368
35	3.4834258 × 10^−17^	7.8 × 10^−12^	2.54 × 10^−8^	0.00000776	0.0005	0.010458206	0.092100486	0.40328124
40	1.9729538 × 10^−19^	2.52 × 10^−13^	2.56 × 10^−9^	0.00000173	0.0001966	0.006176008	0.07162062	0.37138602
45	1.1259474 × 10^−21^	8.22 × 10^−15^	2.6 × 10^−10^	0.000000388	0.000078	0.0036679232	0.055944968	0.34290956
50	6.4643644 × 10^−24^	2.7 × 10^−16^	2.66 × 10^−11^	8.76 × 10^−8^	0.000031	0.0021883948	0.0438608842	0.317304398
55	3.7294886 × 10^−26^	8.86 × 10^−18^	2.72 × 10^−12^	1.99 × 10^−8^	0.00001244	0.00131	0.03449248	0.29415038
60	2.1603519 × 10^−28^	2.94 × 10^−19^	2.8 × 10^−13^	4.52 × 10^−9^	0.000005	0.000788	0.027195754	0.27311594
65	1.2556658 × 10^−30^	9.72 × 10^−21^	2.9 × 10^−14^	0.000000001	0.00000202	0.000474	0.021490666	0.2539335
70	7.319504 × 10^−33^	3.24 × 10^−22^	3 × 10^−15^	2.36 × 10^−10^	0.000000814	0.000286	0.017015502	0.23638314
75	4.277356 × 10^−35^	1.078 × 10^−23^	3.12 × 10^−16^	5.44 × 10^−11^	0.00000033	0.0001734	0.013495322	0.22028128
80	2.5050494 × 10^−37^	3.6 × 10^−25^	3.24 × 10^−17^	1.252 × 10^−11^	1.338 × 10^−7^	0.000105	0.010719656	0.20547284
85	1.4699092 × 10^−39^	1.208 × 10^−26^	3.38 × 10^−18^	2.88 × 10^−12^	5.44 × 10^−8^	0.0000638	0.008526426	0.1918252
90	8.639796 × 10^−42^	4.06 × 10^−28^	3.52 × 10^−19^	6.66 × 10^−13^	2.22 × 10^−8^	0.0000388	0.006790194	0.17922406
95	5.085998 × 10^−44^	1.36 × 10^−29^	3.68 × 10^−20^	1.54 × 10^−13^	0.000000009	0.0000236	0.005413464	0.16756998
100	2.9980656 × 10^−46^	4.58 × 10^−31^	3.86 × 10^−21^	3.56 × 10^−14^	3.68 × 10^−9^	0.0000144	0.0043201898	0.156775866

**Table 2:** Minimal numbers of confirmation blocks for different adversary ratios.

q	0.10	0.15	0.20	0.25	0.30	0.35	0.40	0.45
$P\left(A\left(z\right)\right)<10^{-3}$	7.85976466 × 10^−6^	0.000288	0.000284	0.000748	0.0033027	0.000788	0.00047	0.00099
z	10	10	15	20	25	60	150	540

## Data Availability

Not applicable.

## References

[1] Hellani H., Sliman L., Samhat A.E., Exposito E. (2021). Computing resource allocation scheme for DAG-based IOTA nodes. Sensors.

[2] Garay J., Kiayias A., Leonardos N., Oswald E., Fischlin M. (2015). The bitcoin backbone protocol: Analysis and applications. Advances in Cryptology—EUROCRYPT 2015.

[3] Nakamoto S. Bitcoin: A Peer-to-Peer Electronic Cash System. https://bitcoin.org/bitcoin.pdf.

[4] Saleh F., Jiang W. (2021). Blockchain without waste: Proof-of-Stake. The Review of Financial Studies.

[5] Number Of Orphaned Blocks. https://www.blockchain.com/charts/n-orphaned-blocks.

[6] Rosenfeld M. (2014). Analysis of Hashrate-Based Double Spending.

[7] Pinzón C., Rocha C. (2016). Double-Spend attack models with time advantange for bitcoin. Electron. Notes Theor. Comput. Sci..

[8] Grunspan C., Pérez-Marco R. (2018). Double spend races. Int. J. Theor. Appl. Financ..

[9] Kovalchuk L., Kaidalov D., Nastenko A., Rodinko M., Shevtsov O., Oliynykov R. (2020). Decreasing security threshold against double spend attack in networks with slow synchronization. Comput. Commun..

[10] Proof of Stake Instead of Proof of Work. https://bitcointalk.org/index.php?topic=27787.

[11] Monrat A.A., Schelén O., Andersson K. (2019). Survey of blockchain from the perspectives of applications, challenges and opportunities. IEEE Access.

[12] King S., Nadal S., PPCoin: Peer-to-Peer Crypto-Currency with Proof-of-Stake Self-Published Paper, 19 August 2012. https://decred.org/research/king2012.pdf.

[13] Kiayias A., Russell A., David B., Oliynykov R., Katz J., Shacham S. (2017). Ouroboros: A Provably Secure Proof-of-Stake Blockchain Protocol. LNCS Advances in Cryptology, Proceedings of the CRYPTO 2017: 37th Annual International Cryptology Conference, Santa Barbara, CA, USA, 20–24 August 2017, Part I.

[14] David B., Gazi P., Kiayias A., Russell A. (2017). Ouroboros Praos: An Adaptively-Secure, Semi-Synchronous Proof-of-Stake Protocol. Cryptology ePrint Archive: Report 2017/573. https://eprint.iacr.org/2017/573.

[15] Badertscher C., Gazi P., Kiayias A., Russell A., Zikas V. (2018). Ouroboros Genesis: Composable Proof-of-Stake Blockchains with Dynamic Availability. Cryptology ePrint Archive: Report 2018/378. https://eprint.iacr.org/2018/378.

[16] Badertscher C., Gazi P., Kiayias A., Russell A., Zikas V. (2019). Ouroboros Chronos: Permissionless Clock Synchronization via Proof-of-Stake. Cryptology ePrint Archive: Report 2019/838. https://eprint.iacr.org/2019/838.

[17] Wang W., Li Z., Li H. (2020). Hybrid consensus algorithm based on modified proof-of-probability and DPoS. Future Internet.

[18] Gilad Y., Hemo R., Micali S., Vlachos G., Zeldovich N. (2017). (MIT Computer Science and Artificial Intelligence Laboratory (MIT SCAIL), Cambridge, MA, USA). Algorand: Scaling Byzantine Agreements for Cryptocurrencies. Cryptology ePrint Archive: Report 2017/454. https://eprint.iacr.org/2017/454.

[19] Daian P., Pass R., Shi E. (2016). (Cornell University, Cornell Tech, Ithaca, NY, USA). Snow White: Robustly Reconfigurable Consensus and Applications to Provably Secure Proof of Stake. Cryptology ePrint Archive: Report 2016/919. https://eprint.iacr.org/2016/919.

[20] Longo R., Podda A.S., Saia R. (2020). Analysis of a consensus protocol for extending consistent subchains on the bitcoin blockchain. Computation.

[21] Gaži P., Kiayias A., Zindros D. Proof-of-Stake sidechains. Proceedings of the 2019 IEEE Symposium on Security and Privacy (SP).

[22] Li W., Andreina S., Bohli J.-M., Karame G., Garcia-Alfaro J., Navarro-Arribas G., Hartenstein H., Herrera-Joancomartí J. (2017). Securing Proof-of-Stake Blockchain Protocols. Data Privacy Management, Cryptocurrencies and Blockchain Technology.

[23] Pradhan N.R., Singh A.P. (2021). Smart Contracts for Automated Control System in Blockchain Based Smart Cities. J. Ambient. Intell. Smart Environ..

[24] Raj A., Maji K., Shetty S.D. (2021). Ethereum for Internet of Things security. Multimed. Tools Appl..

[25] Feller W. (1970). An Introduction to Probability Theory and its Applications.

[26] Paris R.B. Chapter 8 Incomplete Gamma and Related Functions. Digital Library of Mathematical Functions. https://dlmf.nist.gov/8.

[27] Kovalchuk L., Rodinko M., Oliynykov R., Kaidalov D., Nastenko A. Probability of double spend attack for network with non-zero synchronization time. Proceedings of the 21st Central European Conference on Cryptology (CECC ’2021).

